# Charity Dinners

**Published:** 1891-05-09

**Authors:** 


### PASSING TOPICS.

### CHARITY DINNERS.

THE festival season is in full swing. Unaffected by those climatic conditions which advance or retard outside vegetation, and in the present year have nearly succeeded in effacing spring altogether from its place in Nature's calendar, the gathering-in of Charity's harvest proceeds apace, and many an institution which sowed in faith a short two or three months ago, has already garnered an abundant yield, and has entered upon a period, all too brief it may be, of peace and plenty.

Cynical moralists are prone to deal contemptuously with the charity dinner, and it has been made the occasion for jibes and jeers innumerable. In regard to this, as other matters of common occurrence, a great deal of ignorance is displayed. The objective point of the joke or the sneer seems to be that before an Englishman can be rendered willing to put his hand into his pocket he must have eaten and drunk enough to have acquired a sort of Dutch benevolence, and that while the victim is under the influence of this spurious sentiment, and altogether quit of such reason and good sense as he can upon other occasions command he is subjected to the somewhat unscrupulous eloquence of a Chairman who is a master of pathetic art, and is compelled by the exercise of a sort of hypnotism to sign a cheque for a sum much in excess of what he is willing to give if, indeed, at a time of perfect sobriety, he would be willing to give anything whatever.

It will, perhaps, surprise moralists of this order to be told that their argument starts from premisses absolutely false. No doubt if we went back to the very earliest of charity dinners we might find that their originator—a great man whose name ought to have been preserved—was well enough acquainted with human nature to entertain a shrewd conviction that self complacency and charity are nearly related, but, as a matter of fact, in these less sentimental and less heavily feeding times, the invitation to a charity dinner, so far from expressing a generally soothing and refreshing influence, is, in a majority of cases, unmistakeably irritating; moreover, many of the chief givers sign their cheques before, and not after dinner, and many more do not dine at all at the charity table.

The abstract and inexperienced reasoner will be disposed to ask, Why, then, go to the expense of a dinner? Well, it does not do to be either abstractive or even strictly logical if you want to be accurate. It is always a pity, though it is often done, to arrive by correct methods at false conclusions, and however thoroughly facts may demonstrate, that the actual eating of the dinner is unnecessary to the generation of generous impulses, we have, upon the other hand, undoubted evidence to show that the premonitory rattling of the plates and the promise of courses to come exercise a magic power we can neither explain nor provide a substitute for. The statement of one hospital at least has been given upon oath to the effect that it is of no use to ask its supporters to make pretence of dinner and to subscribe, just as though it were all real. If any one still doubts that the dinner is the very essence and vitaliser of the effort, let him put the matter to the test. Let him issue a genuine appeal with a genuine chairman, stewards, toasts, subscription lists, and a make-believe menu. Then let him await the result. It may be interesting, but it will scarcely prove substantial.

AGRICOLA.

